# Both hot- and thermoneutral-water immersion reduce 24-h blood pressure in people with hypertension: A randomized crossover study

**DOI:** 10.1080/23328940.2025.2465025

**Published:** 2025-02-27

**Authors:** Brendon H. Roxburgh, Holly A. Campbell, James D. Cotter, Michael J.A. Williams, Kate N. Thomas

**Affiliations:** a School of Physical Education, Sport and Exercise Sciences, University of Otago, Dunedin, New Zealand; b Department of Surgical Sciences, Dunedin School of Medicine, University of Otago, Dunedin, New Zealand; c HeartOtago, University of Otago, Dunedin, New Zealand; d Department of Medicine, Dunedin School of Medicine, University of Otago, Dunedin, New Zealand

**Keywords:** Water immersion, thermoneutral, ambulatory blood pressure, hypertension, heat therapy

## Abstract

The objective of this study was to characterize the 24-h blood pressure response following an acute hot-water immersion exposure, specifically examining the effect of immersion duration and water temperature, in people with hypertension. Sixteen participants (11 females; 62 ± 7 y; 31.7 ± 7.5 kg.m^−2^) with hypertension (taking at least one anti-hypertensive medication) completed four randomized sessions: 1) 20-min and 2) 40-min hot-water immersion (40°C), 3) 40-min thermoneutral immersion (36.5°C), and 4) control (seated, no immersion). Blood pressure, heart rate, perceptual and affective responses were recorded throughout exposure. Immediately after exposure, participants were fitted with an ambulatory blood pressure monitor for the following 24 h. Twenty-four-hour SBP was 7 mm Hg lower (95% CI: −11, −2; *p* = 0.001) after the 40-min hot-water immersion and 6 mm Hg lower after both the 20-min hot-water immersion (−10, −1; *p* = 0.006) and 40-min thermoneutral immersion (−9, −3; *p* = 0.002) compared to control; these effects were similar across nighttime (i.e. 10 pm−6 am) and daytime periods. Twenty-four-hour DBP was not statistically different across any exposure (*p* = 0.093). The percentage of 24 h in target range for SBP (110–130 mm Hg) more than doubled (39% vs. 18%) following the 40-min hot-water immersion, compared to control. Clinically meaningful decreases in systolic blood pressure were evident with hot-water immersion in people with hypertension; these effects were present irrespective of immersion duration (i.e. 20 min vs. 40 min) or water temperature (36.5°C vs 40°C).

## Lay summary

- Systolic blood pressure was 7 mm Hg lower in the 24 h after the 40-min hot-water immersion and 6 mm Hg lower after both the 20-min hot-water immersion and 40-min thermoneutral immersion, compared to control (no immersion).
- These effects were similar across nighttime (i.e. 10 pm-6 am) and daytime periods.
- The percentage of 24 h in target range for systolic blood pressure (110–130 mm Hg) more than doubled (39% vs. 18%) following the 40-min hot-water immersion, compared to control.

## Introduction

Hypertension is a prevalent condition worldwide, significantly increasing the risk of developing heart, brain, and kidney diseases [1,2]. Hypertension burden has increased steadily over the last decade, with approximately half of adults in the United States diagnosed with the condition and a further 10–15% remain undiagnosed [3,4]. However, even small reductions in resting blood pressure are clinically meaningful; a 5 mm Hg decrease in systolic blood pressure is associated with a 10% decrease in the risk of having a major cardiovascular event, in people with or without diagnosed cardiovascular disease (CVD) [5]. Similarly, a 10 mm Hg reduction is associated with a 20% relative risk reduction in major cardiovascular events and an almost halving of the risk of stroke [6,7]. Lifestyle interventions (e.g. physical activity, dietary modification) are recommended first-line treatments for hypertension [8]; however, pharmaceuticals are often favored by clinicians due to lack of patient contact time (i.e. ease of prescription), lack of confidence in counseling patients on exercise, lack of reimbursement and perceived greater likelihood of adherence [9]. Problematically, medication non-adherence is prevalent (up to 50%) and side effects are not uncommon [10,11]; for many, the side effects are more bothersome than the asymptomatic condition they’re being treated for. Moreover, approximately 10–20% of people prescribed maximally tolerated doses of at least three different classes of antihypertensive medication (including a diuretic) cannot achieve target blood pressure [12]. Effective anti-hypertensive therapies that address all aspects of health and promote adherence are needed to improve health outcomes.

Heat therapy is centuries old, historically used by many cultures and civilizations as a form of healing [13,14]. Recent research in Western medicine has reported that it improves cardiovascular health and is associated with a 50% reduction in the risk of stroke and cardiovascular disease mortality [15–18]; part of the lowering of cardiovascular risk may be due to the impressive hypotensive effects of hot-water immersion. Randomized controlled studies have shown reproducible acute reductions in blood pressure, persisting for up to 3-h following a single exposure [19–23]. With repeated exposure (i.e. 3–5 sessions/wk for 12-wk), these hypotensive effects still occur acutely and superimpose on an adaptive reduction that persists for at least a number of days following the previous exposure [22–26]. A sub-analysis of another recently completed trial of 17 patients with hypertension (taking at least one anti-hypertensive medication) revealed a 14 mm Hg and 5 mm Hg decrease in resting systolic and diastolic blood pressure across 12 wk of regular hot-water immersion and calisthenics [23]. Also encouraging was a 98% attendance rate for prescribed heat therapy sessions and the absence of side effects.

Ambulatory blood pressure eliminates many sources of bias associated with blood pressure assessment. As a result, ambulatory blood pressure is the most robust and clinically prognostic assessment of blood pressure. Heat therapy has the potential as an effective anti-hypertensive treatment, but to our knowledge, no research has specifically investigated its effect on 24-h blood pressure in people with hypertension. Moreover, if hot-water immersion is to be used as an adjunct therapy in hypertension management, dose response data are required, including effects of parameters such as the water temperature, duration of immersion, and immersion depth, on ambulatory blood pressure. Therefore, the purpose of this study was to compare blood pressure responses to two such parameters; water temperatures and durations of hot-water immersion. We tested the hypothesis that 20-min or 40-min exposures in hot-water (i.e. 40 °C) or a 40-min exposure in thermoneutral water (i.e. 36.5 °C) would lower 24-h blood pressure to a greater extent, than no exposure.

## Methods

### Ethical approval

All participants provided informed and written consent prior to study participation. This study received ethical approval from the Health and Disability Ethics Committee (2022/EXP/12392), and the study conformed to the standards set by the Declaration of Helsinki. The trial was prospectively registered with the Australian and New Zealand Clinical Trials Registry (ACTRN12622000720718).

### Participants

Seventeen participants (11 women; 62 ± 7 y) with hypertension (i.e. prescribed and regularly taking at least one anti-hypertensive medication) were recruited from the community in Dunedin, New Zealand. All participants were aged 35 y or older, were not pregnant, and had been regularly taking their anti-hypertensive medication for at least 3 months without titration. Participants were not eligible if they had unstable angina, recent myocardial infarction (<3 months ago), history of orthostatic or heat intolerance or any other medical condition deemed a significant risk to study participation. Pre-menopausal women were not excluded; however, all women in the study were post-menopausal and not taking hormone replacement therapy.

### Experimental design

This was a 4 × 4 repeated measures crossover design study. The four exposures were as follows: 1) 20-min hot-water immersion (40.0 ± 0.2 °C), 2) 40-min hot-water immersion (40.0 ± 0.1 °C), 3) 40-min thermoneutral-water immersion (36.7 ± 0.3°C), and 4) 40-min control (no exposure). The order for the four exposures was randomized using a 4 × 4 Latin square design. A minimum 4-d washout period was required between exposures and session time of day was matched within participants.

### Experimental protocol

On the first experimental visit, participants gave consent, underwent a brief health history interview, and height (stadiometer) and body composition (via bioimpedance analysis; InBody 230, InBody, Seoul, South Korea) were measured [27]. Prior to each of the four exposures, participants were asked to void their bladder, and nude body mass was measured using scales (UC-321, A&D, Tokyo, Japan). Urine specific gravity was measured (Uricon – Atago, Tokyo, Japan) to estimate hydration status. If participants were classified as dehydrated (urine specific gravity > 1.020) they received a fluid bolus of 500 mL. However, this was not required as all participants were adequately hydrated.

A 10-min resting baseline period was performed, in which participants were seated wearing swimwear next to the hot tub, and cardiovascular indices and perceptions were obtained (see Measurements for detail). Participants then completed one of the four exposures (see Exposures for detail), with measurements performed at 5 and 10 min, and each 10 min until finishing the exposure. Participants then exited the pool (or remained seated if control), briefly dried themselves with a towel and returned to the pre-exposure seating position for at least 10 min. Baseline measurements were then repeated. Fluid consumed was measured (scales, Model No. 3010, Salter Electronic, Manchester, UK) to estimate fluid balance during each session. Once dressed, participants were fitted with a 24-h accelerometer and 24-h ambulatory blood pressure monitor.

Participants were asked to abstain from, alcohol and caffeinated beverages 12 h prior, and moderate- or high-intensity physical activity for at least 24 h prior to sessions. Participants were reminded to maintain their normal medication and health regimen as advised by their health practitioner(s); pre-exposure, participants were asked whether they had taken their medication and at what time it was taken, to ensure consistency across exposures. All exposures were performed in the same climate controlled laboratory (22.1 ± 1.7°C; relative humidity: 29 ± 5%).

### Experimental measures

#### Cardiovascular

A beat-to-beat finger photoplethysmography device (Finometer, Finapres Medical Systems, The Netherlands) measured blood pressure continuously. Values were calibrated to resting brachial arterial blood pressure measurement obtained via auscultation with a stethoscope and sphygmomanometer (Welch Allyn DS66, New York, USA), per American Heart Association guidelines [28]. Heart rate was measured from the ventricular depolarization by a chest strap (Polar, Finland). Cardiovascular indices were sampled at 4 kHz on an analog-to-digital data acquisition system (PowerLab-8/30, ADInstruments, Dunedin, New Zealand) and recorded on Labchart software (LabChart v.7.3.7, ADInstruments, Dunedin, New Zealand) for analysis.

#### Psychophysiological status

During each exposure, participants rated their feeling (+5 to −5, very good – very bad) [29], thermal sensation (1 to 13, unbearably cold – unbearably hot) and thermal discomfort (1 to 10, comfortable – extremely uncomfortable; adapted from [30]).

#### Twenty-four-hour ambulatory blood pressure monitoring

Ambulatory blood pressure monitoring was performed across the 24 h following each exposure using a brachial cuff-based automated oscillometric device (Oscar 2, SunTech Medical, North Carolina, USA) [31]. Participants were fitted with an appropriately sized cuff on the left arm. The device was programmed to record blood pressure every 30 min for the first 3 h post-exposure, then hourly thereafter. During ambulatory blood pressure measurements, participants were asked to limit disturbances (i.e. telephone talking etc.), avoid having a full bladder, and to be seated upright (if feasible; supine whilst sleeping) and without legs crossed during all measurements. During the initial 24-h monitoring period participants maintained a 24-h food and activity diary; participants were asked to replicate these following the remaining three exposures.

#### Twenty-four-hour physical activity assessment

Participants were asked to maintain their normal habitual activity (and avoid formal exercise) following each exposure. An accelerometer (activPAL3c, Glasgow, Scotland) was fitted to the anterior mid-thigh to assess physical activity patterns during the 24-h blood pressure monitoring period.

### Adverse events

An adverse event was defined as a symptom or medical occurrence during an exposure (e.g. orthostatic hypotension).

### Exposures

#### Twenty-min hot-water immersion

Participants sat upright in 40°C water for 20 min, with a depth approximately nipple height. Both arms were supported at the height of the right atrium on a floating support (i.e. not immersed).

#### Forty-min hot-water immersion

To examine the effect of immersion duration, participants were immersed in 40°C water as per above, for 40 min.

#### Forty-min thermoneutral water immersion

To delineate any effect of heat from the effect of water immersion *per se*, participants immersed in 36.5°C water as per above, for 40 min.

#### Control

To provide a non-exposure reference, following 10 min of pre-exposure seated rest, participants remained seated for a further 50 min to simulate the 40 min exposure and post-exposure recovery period.

### Sample size

Based on data from Roxburgh et al. [23], to detect a clinically significant difference between exposures (i.e. 5 mm Hg) and assuming a power of 0.9 and alpha of 0.05, a sample size of 12 was required; to account for attrition, we recruited 16 participants. GPower software (v. 3.1.9.6) was used for calculations.

### Data and statistical analysis

Accelerometer data were downloaded from the device and analyzed using manufacturer software (PALbatch v8.10, PAL Technologies, Glasgow, Scotland). All accelerometer data were valid. Ambulatory blood pressures were downloaded and analyzed [31–33]. On average across all conditions, 27/29 ambulatory blood pressure recordings (per recording) were valid. A single exposure was repeated for two participants, due to achieving less than 70% valid ambulatory blood pressure recordings across 24 h [31]. Nighttime blood pressure was defined as the mean of measurements taken between 10 pm and 6 am [32]. Time in the therapeutic range was calculated as the percentage of systolic blood pressure measures that were between 110 mm Hg and 130 mm Hg [33].

Descriptive data are expressed as mean ± standard deviation, median [25th, 75th quartiles], or number (proportion). A repeated-measures analysis of variance (ANOVA) was used to compare differences in continuous post-exposure variables across the four exposures. Acute responses were compared using a two-way ANOVA. Comparisons of interest are reported as mean differences with 95% confidence interval [lower limit, upper limit]. Non-normally distributed data, ratio data (i.e. time in therapeutic range), and ordinal data (i.e. psychophysiological scales) are presented as median and compared using a Friedman test, with Dunn’s test used to isolate any differences between groups; comparisons were made only at baseline, 20 min and 40 min for psychophysiological scales to avoid multiplicity. Simple linear regression was used to assess the effect of resting blood pressure on 24-h blood pressure response. GraphPad Prism (version 10.1.1, Boston, USA) was used for statistical analysis and figures.

## Results

Sixteen participants completed all four exposures (Table 1) across June 2022 to December 2022. One participant withdrew from the study after two exposures due to personal reasons; their data were not analyzed. Nine participants were meeting physical activity guidelines [34], four were active but did not reach guideline levels, and two were not engaging in any leisure-time physical activity. Step count in the 24 h following each exposure was not different (*p* = 0.908).

**Table 1.** Descriptive statistics of participants. Data are mean (SD), median (range), or as an absolute number with the percentage (%) of the whole.

Variable	Mean (SD)/n (%)
Age (y)	62 (7)
Male/female	5 (31%)/11 (69%)
Height (cm)	165 (8)
Body mass (kg)	87 (19)
BMI (kg.m^−2^)	31.7 (7.5)
Muscle mass (kg)	29 (7)
Fat free mass (kg)	53 (12)
Body fat (%)	38 (10)
**Ethnicity**	
New Zealand European	13
New Zealand Māori	1
Cook Island Māori Botswana	1 1
European	1
**Comorbidity** Asthma Chronic kidney disease Coronary artery disease Dyslipidaemia Obesity	1 (6%) 0 1 (6%) 2 (13%) 7 (44%)
Pre-diabetes/diabetes	2 (13%)
Osteoarthritis	9 (56%)
**Anti-hypertension medication** Number of anti-hypertensive drugs ACE inhibitor Beta-blocker Calcium channel blocker ARB ARB + diuretic	1 (1–3) 4 (25%) 1 (6%) 5 (31%) 8 (50%) 3 (19%)
Vasodilator	2 (13%)

BMI = body mass index; ACE = angiotensin-converting-enzyme; ARB = Angiotensin receptor blocker.

### Ambulatory blood pressure monitoring

**Figure 1.**
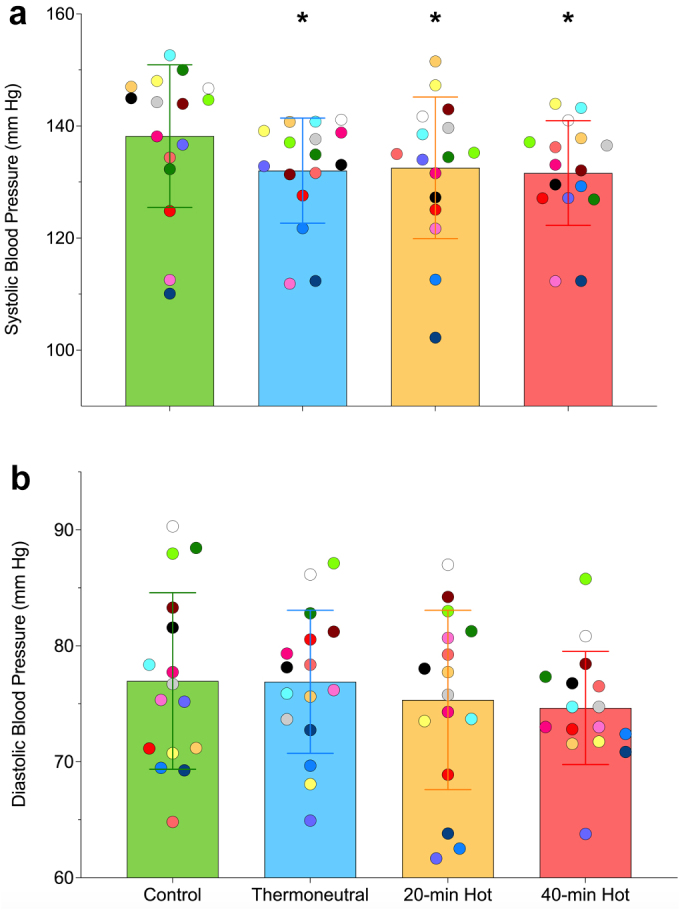
Ambulatory systolic blood pressure (a) and diastolic blood pressure (b) by exposure. Results from individual participants (each symbol represents an individual participant’s mean 24-h blood pressure across the four exposures; error bars indicate 95% CI) are presented for each exposure.*p < 0.05 vs. control. *n* = 16 for all.

**Figure 2.**
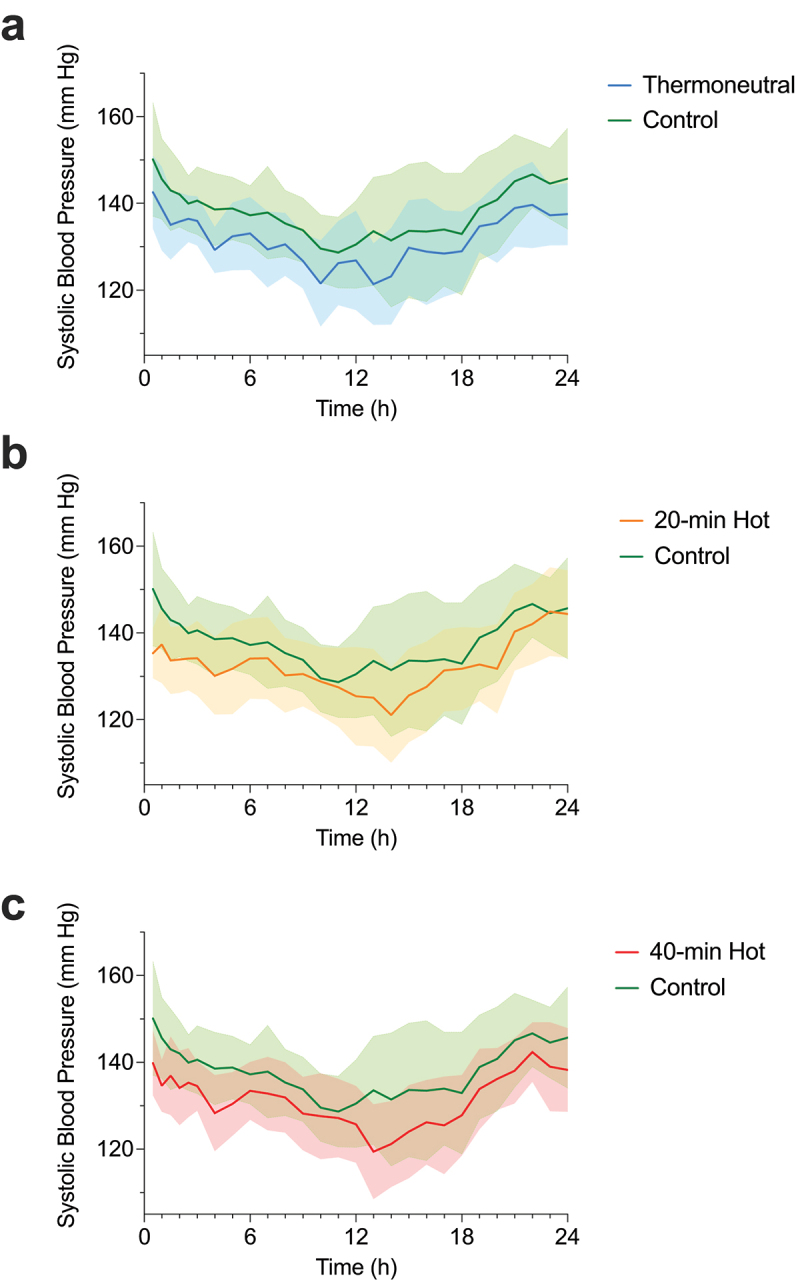
Ambulatory systolic blood pressure (and shaded 95% CI) across 24-h following thermoneutral (a), 20-min (b; 20-min Hot) and 40-min exposures (c; 40-min Hot), all vs. control exposure. *n* = 16 for all.

Twenty-four-hour systolic blood pressure was 7 mm Hg lower (95% CI: −11, −2; *p* = 0.001) after the 40-min hot-water exposure, and 6 mm Hg lower after both the 20-min (−10, −1; *p* = 0.006) and 40-min thermoneutral exposures (−9, −3; *p* = 0.002) compared to control (Table 2, Figures 1 & 2). No other differences in 24-h systolic blood pressure (*p* ≥ 0.937) or 24-h diastolic blood pressure were evident between exposures. Participants with a higher resting systolic blood pressure experienced a greater reduction across 24 h following 40-min (R^2^ = 0.35, β = −0.259, *p* = 0.016), 20-min (R^2^ = 0.31, β = −0.257, *p* = 0.024), and thermoneutral (R^2^ = 0.26, β = −0.1622, *p* = 0.042) exposure.

**Table 2.** Ambulatory blood pressure across 24-h post-exposure.Blood pressure data are presented as mean (SD) and analyzed with a repeated-measures ANOVA and therapeutic range is presented as median [25th, 75th quartiles] and analyzed using a Friedman Test. * = p < 0.05 vs. control. Mean difference between exposures (95% confidence intervals) is also presented. *n* = 16 for all.

	Control	Thermoneutral	20-min	40-min	P-value	C v TWI	C v 20-min HWI	C v 40-min HWI
*Mean 24-h blood pressure*								
Systolic blood pressure (mm Hg)	138 (13)	132 (9)*	133 (13)*	132 (9)*	**<0.001**	**-6 (−9, −3)**	**-6 (−10, −1)**	**-7 (−11, −2)**
Diastolic blood pressure (mm Hg)	77 (8)	77 (6)	75 (8)	75 (5)	0.093	0 (−3, 3)	−2 (−5, 1)	−2 (−5, 1)
Time in therapeutic range (%)	18 [13,33]	34 [25,35]*	30 [13,36]*	39 [22,37]*	**0.009**			
*Mean daytime blood pressure*								
Systolic blood pressure (mm Hg)	144 (13)	137 (10)*	137 (12)*	137 (9)*	**<0.001**	**-7 (−11, −3)**	**-7 (−12, −2)**	**-8 (−13, −2)**
Diastolic blood pressure (mm Hg)	77 (8)	77 (6)	75 (8)	75 (5)	0.094	0 (−3, 3)	−2 (−2, 5)	−2 (−1, 6)
*Mean nighttime blood pressue*								
Systolic blood pressure (mm Hg)	128 (17)	122 (13)*	122 (13)	121 (13)*	**0.032**	**-6 (−10, −1)**	**-6 (−12, 0)**	**-7 (−14, −1)**
Diastolic blood pressure (mm Hg)	68 (9)	69 (6)	67 (9)	67 (7)	0.622	1 (−3, 5)	−1 (−5, 4)	−1 (−5, 4)

TWI, thermoneutral-water immersion; HWI, hot-water immersion; Daytime = 6 am − 10 pm; Nighttime = 10 pm − 6 am; time in therapeutic range = percentage of 24 h that systolic blood pressure is 110–130 mm Hg.

Daytime systolic blood pressure was 8 mm Hg lower (−13, −2; *p* = 0.005) after the 40-min hot-water exposure, and 7 mm Hg lower after both the 20-min (−12, −2; *p* = 0.004) and 40-min thermoneutral exposures (−11, −3; *p* = 0.001) compared to control (Table 2). No other differences in daytime systolic blood pressure (*p* ≥ 0.986) or daytime diastolic blood pressure were evident between exposures.

**Figure 3.**
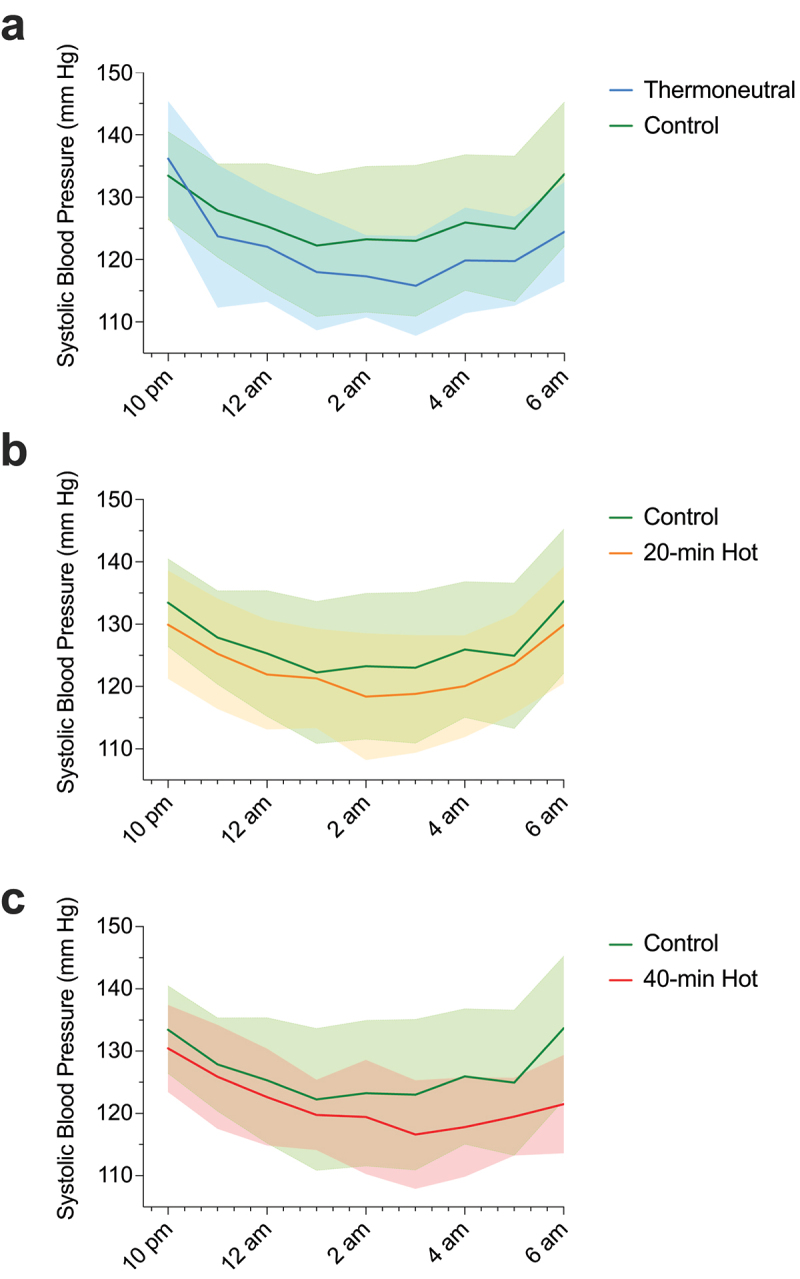
Ambulatory night time (10pm-6am) systolic blood pressure (and shaded 95% CI) following thermoneutral (a), 20-min (b; 20-min Hot) and 40-min exposures (c; 40-min Hot), all vs. control exposure. *n* = 16 for all.

Similarly, nighttime systolic blood pressure was 7 mm Hg lower (−14, −1; *p* = 0.030) after the 40-min hot-water exposure and 6 mm Hg lower after the 40-min thermoneutral exposure (−10, −1; *p* = 0.014), compared to control (Table 2 & Figure 3); the 6 mm Hg mean difference in nighttime systolic blood pressure between the 20-min and control exposure was not statistically significant (−6 mm Hg: −12, 0; *p* = 0.059). There were no other differences in nighttime systolic blood pressure between exposures (*p* ≥ 0.511) or nighttime diastolic blood pressure (*p* = 0.622).

The duration that systolic blood pressure was within therapeutic target range was more than doubled following the 40-min exposure (39% vs. 18%; rank sum difference: +17.5%; *p* = 0.002), compared to control (Table 2), and was higher also following the 20-min (+18%; *p* = 0.014) hot-water and thermoneutral (+22.5%; *p* = 0.002) exposures compared to control.

### Acute response

**Figure 4.**
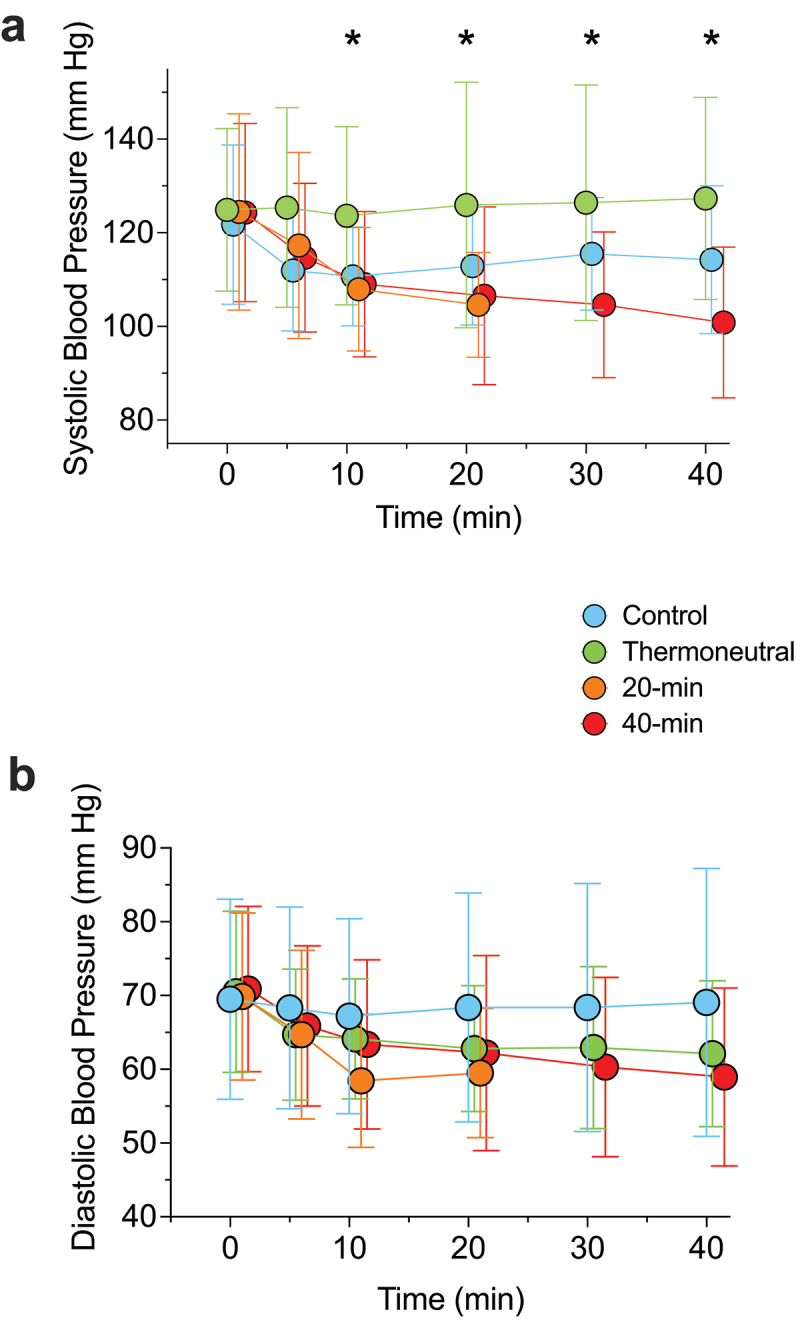
Systolic blood pressure (a) and diastolic blood pressure (b) during control, 40-min thermoneutral- (36.5°C), 20-min hot- (40°C) and 40-min hot-water (40°c) exposures. *n* = 16 for all. * = significant difference (i.e. *p* < 0.05) between thermoneutral, 20-min at 40°C and 40-min at 40°C vs. control.

Systolic blood pressure decreased upon immersion in water and was below that of the control exposure from 10 min onwards (*p* ≤ 0.041; Figure 4); the largest difference was at 40-min during the hot exposure (vs. control: −27 mm Hg; 14 to 39; *p* = 0.004). From 20-min onwards, systolic blood pressure continued to decrease during hot-water immersion relative to the thermoneutral exposure (30-min: −10 mm Hg; 3 to 17, *p* = 0.007; 40-min: −13 mm Hg; 7 to 20, *p* = 0.001). Diastolic blood pressure trended lower during all three immersion exposures, compared to control, but these were not statistically significant (*p* ≥ 0.080; Figure 4).

**Figure 5.**
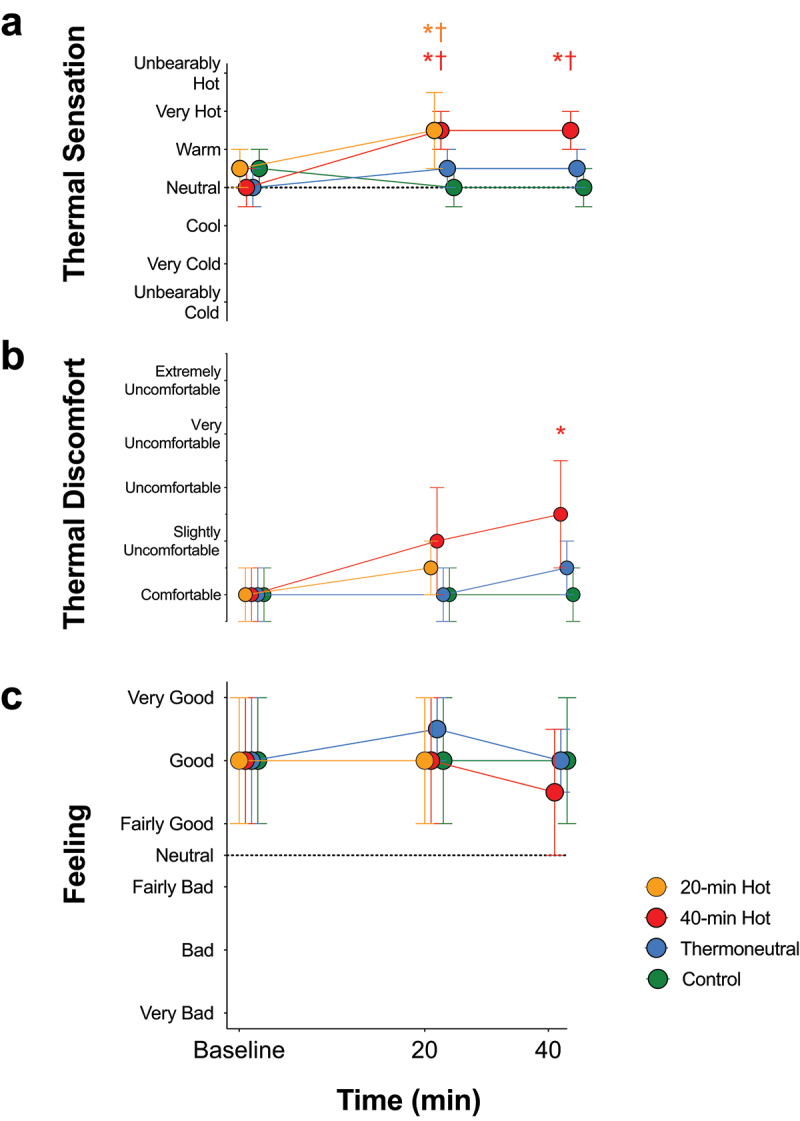
Thermal sensation (a), thermal discomfort (b) and feeling scale (c) responses during control, 40-min thermoneutral- (36.5°C), 20-min hot- (40°C) and 40-min hot-water (40°C) exposures. *n* = 16 for all. * = significant difference (i.e. *p* < 0.05) vs. control; † = significant difference vs. thermoneutral.

Median perceived body temperature (i.e. thermal sensation; *p* = 0.173), thermal discomfort (*p* = 0.650) and feeling (*p* = 0.762) were not different between exposures at baseline (Figure 5). By 20 min of immersion, median perceived body temperature was “hot” in both hot-water exposures (*p* < 0.001 for both vs control), and neither thermal discomfort nor feeling differed between exposures (*p* ≥ 0.191). By 40-min the hot-water immersion was thermally “uncomfortable” (*p* ≤ 0.002 vs thermoneutral and control), but feeling remained similar between exposures (*p* = 0.126).

### Safety and adverse events

Participants completed all sessions without incident, with no adverse events occurring.

## Discussion

This study reports several novel findings: 1) hot-water immersion elicited a substantial and clinically meaningful decrease in systolic blood pressure in people with hypertension; 2) this was irrespective of duration of exposure (i.e. 20 min vs. 40 min) or water temperature (36.5°C vs 40°C); 3) the percentage of time systolic blood pressure was in target range (i.e. 110–130 mm Hg) in the 24-h following a single exposure was approximately doubled; and 4) no reduction in ambulatory diastolic blood pressure was evident.

Following each of the three immersion protocols, the ambulatory systolic blood pressure was 6–7 mm Hg lower in the following 24 h compared to control; the magnitude of this response was consistent across day and night. Diastolic blood pressure was not different between exposures. Importantly, systolic blood pressure is recognized as having the most clinical significance due to its stronger correlation with cardiovascular events and impact on other organs (e.g. brain, kidneys) [38,39]. Specifically, large meta-analyses report that a 5–10 mm Hg decrease in systolic blood pressure is associated with a 10–20% decrease in major cardiovascular events, in people with or without CVD [5–7].

Time in the therapeutic range is a novel index of hypertension management, reflecting how well blood pressure is controlled across a substantial monitoring period [33]. The percentage of time in therapeutic range was approximately doubled following any immersion, compared to the control exposure. The Veterans study (approximately 700,000 participants) reported that 10-y cardiovascular risk and mortality rate is 50% higher in those with a systolic blood pressure time in therapeutic range of 0–25%, compared to >25–50% [33]; our 40-min hot-water immersion exposure moved 12/16 participants into this lower risk category. In another study, for every ~10% increase in time in the therapeutic range, the risk of first major cardiovascular event is reduced by more than 20% [40]. Although this study only looked at the acute (24 h) effect, previous work has highlighted that the acute   post-exposure hypotensive response does not diminish with repeated exposure across 12 wk [23]. Furthermore, this acute response superimposes on an underlying adaptive effect that has been demonstrated by ourselves and others in normotensive individuals [23–26]. Therefore, if hot-water immersion is practiced regularly as part of a healthy lifestyle (i.e. alongside regular exercise and a healthy diet), these repeated hypotensive periods likely afford clinical benefit.

A somewhat surprising finding in this study was that the thermoneutral immersion reduced 24-h blood pressure to a similar extent to the hot-water immersion; however, this is supported in the literature. Although the acute systolic blood pressure responses during thermoneutral immersion are conflicting [22,41,42], Kissling et al. [22] reported a similar hypotensive effect 24-h following hot or thermoneutral immersion that was maintained after 5 d of repetition. Brunt et al. [26] have also compared hot-water (40.5°C) vs. thermoneutral-water (36°C) immersion in a repeated design in sedentary, young participants. Across 8 wk, systolic blood pressure was reduced similarly between groups (−4 mm Hg and −3 mm Hg; *n* = 10 per group). These reductions would likely be larger in those with the highest resting blood pressure, based on previous hot-water immersion and exercise literature [23,43].

Contributing mechanisms for this hypotensive effect are potentially multifactorial and complex. Immediately upon immersion in chest-deep water, the central blood volume rises ~700 mL, with a concurrent dose response increase in stroke volume with increasing water temperature [20,44,45]. Despite these centrally mediated changes, systolic blood pressure remains unaffected in young adults, with progressive reductions in total peripheral resistance with increasing water temperature (50–65%) offsetting the increase in preload and myocardial contractility [44]; it is unclear how this response differs in older adults or those with hypertension, or how long these reductions in total peripheral resistance persist. With the increased cardiac output and concomitant reduction in sympathetically mediated constrictor tonic activity, there is an increase in peripheral blood flow and antegrade vascular shear stress which results in endothelium-mediated nitric oxide release, triggering arterial vasodilation similar to that of high-intensity exercise [46,47]. Brunt et al. [26] reported improved vascular function (i.e. flow-mediated dilation, superficial femoral artery compliance) with 8 wk of hot – but not thermoneutral-water immersion. Furthermore, the lack of change in 24-h diastolic blood pressure (a surrogate for total peripheral resistance) in the present study further implies that the hypotensive benefits may be mediated via other mechanisms, driven by the hydrostatic effects of water, rather than heat *per se*.

Diuresis and natriuresis are additional hydrostatic responses that likely contribute to hypotensive effects [44,48]. The increased venous return during immersion and sustained stretch on the right atrium increases the release of atrial natriuretic peptide [36]; curiously, atrial natriuretic peptide response may be greater during thermoneutral immersion, compared to hot-water immersion [22]. Atrial natriuretic peptide also has a suppressive effect on fluid regulatory hormones such as aldosterone and vasopressin and increases in prostaglandin E; this increases sodium and water excretion and therefore reduces fluid volume *during* immersion. Atrial natriuretic peptide also has mild vasodilatory properties. It is plausible that these diuretic and natriuretic effects are followed by a supercompensation in plasma volume such as occurs in the post-exercise hypotensive response. The ensuing reduction in resting heart rate, increase in parasympathetic activity and arterial baroreceptor resetting are likely part of a multifactorial cascade contributing to the hypotensive effects of water immersion [37].

Side effects are common with anti-hypertensive medications [10,11]. Reassuringly, the hypotensive effects observed in this study, albeit in a modest sample, occurred without any associated side effects or adverse events. Additionally, although hot-water immersion became more uncomfortable with prolonged exposure, psychophysiological responses remained similar and favorable across all immersion conditions. These findings indicate that users are likely to tolerate and engage in this form of therapy, as the absence of physiological side effects and modest perceptual and affective responses (thermal discomfort and feeling state) do not affect the overall experience.

### Perspectives and limitations

This study provides valuable insight into the effects of hot-water immersion on blood pressure in people being treated for hypertension. We would like to raise a number of considerations. A hot tub is a preferred modality in the laboratory setting due to its ease of controlling potential confounding variables (e.g. water temperature, posture, depth). However, they are resource intensive, requiring large volumes of water to be maintained at a hot temperature, limiting their accessibility and equitability as a form of therapy. Importantly though, the findings of this study suggest that as an adjunct anti-hypertensive therapy, water temperature may not need to be maintained at ~ 40°C, thereby reducing the heating requirements. Future work should explore the effect of immersion depth and examine whether less resource-intensive modalities elicit similar effects, such as a home bath or lower-leg immersions in a small tub or bucket.

Another notable observation was the variability in response to each exposure (Figure 1). Previous research on thermoneutral immersion has reported blunted diuresis and less reduction in vasopressin concentration in endurance-trained compared to untrained individuals [35,49]. Furthermore, older individuals have reduced cardiac baroreflex sensitivity [50–52]; this may contribute to larger hypotensive responses via a reduced capacity or failure to counteract systemic vasodilation [53]. Conversely, aging can also blunt the reduction in vascular resistance in response to heating, which might blunt the hypotensive response [52]. More studies are required to compare acute (especially 12–24 h) and adaptive responses in different populations, particularly regarding how factors such as age and fitness level influence responses to immersion.

Although the study participants were diagnosed with hypertension and taking anti-hypertensive medication, a number of participants in the study cohort had well-controlled blood pressure (SBP range: 110–152 mm Hg). Our analysis showed that those with higher resting systolic blood pressure experienced a greater reduction across 24 h following all immersion exposures. Furthermore, evidence from exercise studies indicates larger acute and adaptive reductions in resting blood pressure for those with higher blood pressures [43]. Higher resting blood pressure is associated with greater natriuresis and larger acute reductions in systolic blood pressure, following thermoneutral immersion [54]. Therefore, the findings in this study may be underreporting the possible effect in people with higher or less well-controlled resting blood pressure. Lastly, we acknowledge the potential for a familiarization effect on blood pressure as participants become more accustomed to the cuff across multiple monitoring periods [55]. However, we are confident that the randomized design effectively distributed any such effect across all four exposures.

## Conclusions

A single session of water immersion reduced systolic blood pressure by ~7 mm Hg in the ensuing 24 h. The magnitude of response was similar across day and night and occurred irrespective of water temperature or immersion duration. We are not suggesting it should replace pharmaceuticals or serve as an alternative to regular physical activity for blood pressure control. However, water immersion likely has complimentary and additive effects alongside these traditional therapies. Future research must explore the mechanisms contributing to these hypotensive effects, and if more ecologically valid and equitable applications of water immersion can elicit similarly beneficial hypotensive effects.
